# Mental health of caregivers for people with dementia and cerebral palsy as a key determinant of caregiver burden: a multivariable analysis

**DOI:** 10.3389/fpubh.2026.1757413

**Published:** 2026-03-04

**Authors:** Alba Sánchez-Gil, Andrea Calleja-Caballero, Fátima Pérez-Robledo, Carlos Martín-Sánchez, María Rodríguez-Lorenzo, María Martínez-Romo, Enrique Pérez-Saez, Pedro Manuel Rodríguez-Muñoz, Cristina Rivera-Picón, Jesus Perez, Juan Luis Sánchez-González

**Affiliations:** 1 National Centre of Reference for People with Alzheimer’s Disease and Other Dementias, Salamanca, Spain; 2 Departamento de Enfermería y Fisioterapia, Instituto de Investigación Biomédica de Salamanca (IBSAL), Universidad de Salamanca, Salamanca, Spain; 3 Master’s Degree in Intervention for People with Alzheimer’s Disease, Faculty of Medicine, University of Salamanca, Salamanca, Spain; 4 Facultad de Enfermería y Fisioterapia, Universidad de Castilla-La Mancha, Toledo, Spain; 5 Facultad de Enfermería y Fisioterapia, Universidad de Castilla-La Mancha, Talavera de la Reina, Spain; 6 Departamento de Medicina, Instituto de Investigación Biomédica de Salamanca (IBSAL), Universidad de Salamanca, Salamanca, Spain

**Keywords:** anxiety, burden, caregiver, depression, mental health, moderation analysis

## Abstract

**Background:**

Informal caregiving plays a vital role in supporting dependent individuals; however, prolonged caregiving is associated with significant physical and psychological strain. Understanding factors associated with caregiver burden is essential for designing effective interventions to protect caregiver health and sustain long-term care systems.

**Objective:**

To examine the associations between psychological, physical, and contextual factors on caregiver burden and to identify variables independently associated with caregiver burden.

**Methods:**

A cross-sectional study was conducted with 73 informal caregivers of people living with dementia or cerebral palsy who required substantial assistance in daily living. Standardized instruments were used to assess caregiver burden (Zarit Burden Interview), anxiety and depression (HADS), mental health and quality of life (SF-36), pain (VAS), and physical activity (IPAQ). Hierarchical multiple regression, mediation, and moderation analyses were performed.

**Results:**

Mental health and anxiety showed the strongest independent associations of caregiver burden (*β* = −0.396, *p* = 0.002; *β* = 0.243, *p* = 0.049, respectively), followed by musculoskeletal pain in the back and lower limbs. Patient-related variables, such as functional dependence or disability, were not directly associated with burden. Mediation analysis showed that mental health did not mediate the dependence-burden link. Moderation analysis did not reveal a statistically significant interaction effect. The final model explained over 60% of the variance in caregiver burden.

**Conclusion:**

Caregiver mental health is a key determinant of perceived burden of caring for people with dementia and cerebral palsy, exerting a stronger influence than patient dependence or physical demands. Interventions should integrate psychological screening and mental health support to prevent caregiver distress and ensure a sustainable informal care.

## 1 Introduction

The progressive demographic aging and the rising prevalence of chronic diseases have led to a sustained increase in the demand for long-term care, particularly within home-based settings (1). In this context, informal caregivers, unpaid individuals who are often family members and assume responsibility for the daily care of persons with functional dependence or chronic illness, have become an essential component of health and social care systems. Their involvement significantly contributes to the sustainability of care for dependent individuals, facilitating the maintenance of care recipients in their usual living environments and reducing the need for institutionalization or formal care services (2, 3). From a public health perspective, informal caregivers represent an invisible yet indispensable pillar in maintaining community health and optimizing overall health system efficiency (4). However, the provision of continuous care without a formal support structure carries substantial consequences for caregiver health (5). This phenomenon, commonly referred to as caregiver burden, is defined as the physical, emotional, social, and economic impact resulting from prolonged caregiving. Caregiver burden manifests as physical fatigue, musculoskeletal pain, functional limitations, social isolation, anxiety, depressive symptoms, and, in severe cases, burnout syndrome (6, 7). Therefore, understanding the factors that determine caregiver burden is a priority for developing effective interventions aimed at promoting caregiver well-being and ensuring the sustainability of informal care.

This study was guided by the Stress Process Model of caregiving, which conceptualizes caregiver outcomes as the result of an interplay between caregiver background/resources, primary stressors anchored in caregiving demands (e.g., care-recipient functional impairment and dependence), secondary strains (e.g., role strains and physical health consequences such as musculoskeletal pain), and psychosocial vulnerability/resources shaping appraisal and adaptation. Within this framework, caregiver burden is understood as a caregiving-specific appraisal of role-related strain. Accordingly, we examined indicators of care demands (dependence/disability and caregiving exposure), physical strain (region-specific pain and physical activity), and psychosocial factors (anxiety/depression and mental health-related quality of life) as jointly contributing correlates of perceived burden, and explored whether mental health operates as a mediator or a moderator in the demand-burden association (8).

Among the conditions that require sustained informal care, dementia and cerebral palsy are particularly notable. Although these conditions differ in etiology and progression, both share the need for constant attention and high levels of physical and emotional demand on the caregiver (9, 10). In dementia, the progressive deterioration of cognitive functions, memory impairments, disorientation, and behavioral changes in the patient generate increasing dependency that requires continuous supervision and intense emotional involvement (11). Caregivers face situations of progressive loss of autonomy and communication, often resulting in anxiety, depression, and emotional exhaustion (12). Moreover, as the disease progresses, assistance with activities, such as mobility, feeding and personal hygiene, becomes increasingly frequent and demanding, contributing further to physical fatigue and musculoskeletal pain in caregivers (13). Cerebral palsy, on the other hand, represents a leading cause of chronic physical disability from early life. Motor limitations, postural abnormalities, and the presence of neurological or sensory comorbidities demand prolonged and physically intensive care (14). Caregivers consistently perform tasks such as mobilization, hygiene assistance, and supervision, which may result in musculoskeletal pain or chronic fatigue. In addition, the emotional burden associated with managing a chronic and irreversible condition, the constant concern for the future of the care recipient, and the need to adapt one’s life to caregiving demands increase psychological vulnerability and the risk of anxiety and depression in caregivers (15, 16).

Despite the extensive literature addressing individual aspects of caregiver burden, few studies have analyzed physical, emotional, and contextual components of caregiving within a single integrated model. This limitation hampers the identification of the most robust predictors of burden and, consequently, the development of targeted prevention and support strategies. Integrating these components is particularly crucial in the public health context, as the cumulative impact of caregiver burden not only affects caregiver well-being, but also the quality of care provided and, by extension, the health outcomes of dependent individuals.

Although dementia and cerebral palsy differ substantially in etiology, age of onset, and life-course trajectory, both conditions frequently entail long-term, high-intensity informal caregiving characterized by marked functional dependence and sustained physical and emotional demands. In the present study, the analytical focus was therefore placed on caregiver-related determinants of burden that are conceptually transversal across diagnoses—such as mental health and musculoskeletal pain—rather than on diagnosis-specific comparisons. This approach allows the identification of common, potentially modifiable factors associated with caregiver burden across different caregiving contexts.

Based on the Stress Process Model, the present study aimed to provide a comprehensive analysis of factors associated with caregiver burden in adult informal caregivers for people with dementia and cerebral palsy by jointly examining psychological, physical, and contextual factors. Specifically, the objectives were threefold:

- To examine the associations between caregiver burden and indicators aligned with the stress-process framework, including primary caregiving demands (care-recipient dependence/disability and caregiving exposure), secondary physical strains (region-specific pain and physical activity), and psychosocial vulnerability/resources (anxiety, depression, and mental health-related quality of life).
- To identify variables independently associated with caregiver burden through hierarchical regression, estimating the relative contribution of caregiving demands, physical strain, and psychosocial factors.
- To explore whether caregiver mental health is consistent with a mediating pathway or a moderating/buffering pattern in the association between care demands (dependence) and perceived burden.

## 2 Methods

### 2.1 Study design

To achieve our objectives, we carried out a descriptive, observational, cross-sectional study, which received approval from the Ethics Committee of the University of Salamanca (Record No. 1066). All procedures adhered to the latest revision of the Declaration of Helsinki (17). Data collection was conducted between January 2025 and July 2025 in Salamanca (Spain).

### 2.2 Study participants

Informal caregivers of individuals diagnosed with dementia (including Alzheimer’s disease and related dementias) or cerebral palsy were recruited to participate in this study. Recruitment was facilitated through a collaboration agreement with the State Reference Centre for the Care of People with Alzheimer’s Disease and Other Dementias (CREA) of the Institute for the Elderly and Social Services (IMSERSO) in Salamanca, and with the Association of Parents of People with Cerebral Palsy and Related Encephalopathies of Salamanca (ASPACE), Spain.

Eligible participants were adults (≥18 years) who acted as the primary informal caregiver of a person with dementia or cerebral palsy and had at least 1 year of caregiving experience in that role. Caregivers were excluded if they were illiterate or otherwise unable to complete the interview process, or if they served as secondary or formal caregivers.

### 2.3 Sampling strategy and recruitment

A non-probability consecutive sampling strategy was used. Participants were recruited among informal caregivers of users attending the collaborating institutions (CREA-IMSERSO and ASPACE) in Salamanca, Spain. Recruitment and data collection took place between January 2025 and July 2025.

Potential participants were contacted by institutional staff via invitation letters and follow-up phone calls. A total of 100 caregivers were contacted, and 80 underwent eligibility screening. Of these, 73 met the inclusion criteria and completed the scheduled interview comprising the final analytical sample (*n* = 73, complete data). The remaining 7 caregivers did not attend the scheduled interview and were excluded from the study.

### 2.4 Evaluations

Each participant was individually invited to a room equipped for testing under controlled environmental conditions (e.g., lighting, temperature, noise). An identification code was assigned to each participant to preserve anonymity and ensure confidentiality. Subsequently, a digital questionnaire comprising three sections was completed: (1) an information sheet outlining the study, including a digital informed consent form; (2) a structured set of questions to gather relevant sociodemographic data (e.g., age, gender); and (3) standardized questionnaires for the assessment of the following study variables:

#### 2.4.1 Caregiver burden

Caregiver burden was assessed using the 22-item Zarit Burden Interview (ZBI), with each item rated on a 5-point Likert scale (0–4), yielding a total score from 0 to 88. Higher scores reflect greater perceived burden. The ZBI evaluates the caregiver’s subjective perception of the impact of caregiving on emotional well-being, physical health, social life, and financial status (18). A cut-off score of ≥57, based on previous validation in Spain, was used to categorise caregivers as experiencing burden. The instrument demonstrated high internal consistency (Cronbach’s *α* = 0.92).

#### 2.4.2 Physical activity

Overall physical activity was assessed using the Spanish version of the International Physical Activity Questionnaire (IPAQ) (19). The IPAQ consists of seven items aimed at evaluating the duration and frequency of physical activity across different intensity levels. Light activity (<600 MET minutes/week) includes actions such as walking at home, at work, or during leisure; moderate activity (600–3,000 MET minutes/week) encompasses tasks like cycling, playing tennis, or carrying light loads; and vigorous activity (>3,000 MET minutes/week) involves high-intensity efforts such as aerobic exercise, digging, or heavy lifting. The questionnaire also records sedentary behavior over the previous week, offering a comprehensive overview of the participant’s physical activity profile. The total physical activity score is calculated by summing the MET minutes/week from vigorous, moderate, and light activity levels (20).

#### 2.4.3 Anxiety and depression

Anxiety and depression were assessed using the Hospital Anxiety and Depression Scale (HADS), a widely recognised and validated instrument for use across various populations (21). The HADS comprises 14 items in total, with seven questions evaluating anxiety (subscale A) and the remaining seven assessing depression (subscale D), each rated on a 4-point Likert scale from 0 to 3. This format yields separate scores for anxiety and depression, allowing for a nuanced assessment of each construct.

All items were scored according to the original instrument guidelines. Responses were coded and summed following the standard HADS scoring procedure, and no additional reverse coding was required beyond that inherent to the scale structure.

#### 2.4.4 Pain

Pain intensity was evaluated using the Visual Analogue Scale (VAS) for pain, administered as a 10-cm horizontal line anchored by “no pain” and “worst pain imaginable” (22). Participants marked the point that best represented their pain intensity at the time of assessment, and the distance from the “no pain” anchor was recorded. Scores were expressed on a 0–10 metric (cm), equivalent to a 0–100 mm VAS.

For descriptive interpretation only, pain intensity was categorized using a commonly applied 0–10 cut-point scheme: mild [1–3], moderate [4–6], and severe [7–10] (23). As cut points may vary across populations and contexts (24), VAS scores were treated as continuous variables in all regression, mediation, and moderation analyses.

#### 2.4.5 Quality of life

Health-related quality of life was evaluated using the Spanish version of the Short Form-36 Health Survey (SF-36), a validated instrument derived from the Medical Outcomes Study (25). The questionnaire comprises 36 items grouped into eight dimensions: physical functioning, role-physical, bodily pain, general health, vitality, social functioning, role-emotional, and mental health. Scores for each dimension range from 0 (worst health) to 100 (best health), with higher scores indicating better perceived health status (26). The Spanish adaptation was conducted following the standardized IQOLA protocol to ensure linguistic and cultural validity (27).

Negatively worded items were reverse-coded prior to score calculation, in accordance with the SF-36 scoring manual. Domain scores were subsequently transformed to a 0–100 scale, with higher scores indicating better perceived health status.

### 2.5 Sample size adequacy (sensitivity analysis)

Given the observational design and consecutive recruitment from a finite pool of eligible caregivers attending the collaborating institutions during the study period, the sample size was determined pragmatically by feasibility. A sensitivity power analysis was conducted to evaluate whether the achieved sample was sufficient to detect associations of interest. With *n* = 73, a two-tailed *α* = 0.05 and 80% power, the study was able to detect a minimum Pearson correlation of approximately |*r*| = 0.32, corresponding to a moderate effect size.

### 2.6 Statistical analysis

Statistical analysis was performed using IBM SPSS Statistics software (version 28). A statistical significance level of *p* < 0.05 was established for all tests.

#### 2.6.1 Preliminary and descriptive analysis

The normality of quantitative variables was assessed using the Kolmogorov–Smirnov test. As several variables showed non-normal distributions, non-parametric procedures were applied for inferential analyses.

Descriptive statistics—including means, standard deviations, frequencies, and percentages—were calculated to summarize sample characteristics.

#### 2.6.2 Relationships between variables

The associations among continuous variables were explored using Spearman’s rho correlations, given the non-normality of several distributions.

Correlation coefficients were interpreted according to Cohen’s guidelines (|*r*| = 0.10 small, 0.30 moderate, 0.50 large). Significant correlations guided the selection of variables for inclusion in the subsequent regression and mediation models.

#### 2.6.3 Hierarchical regression analysis

Variables were entered in theoretically meaningful blocks to examine the incremental contribution of physical and psychological factors beyond patient-related characteristics. A hierarchical multiple linear regression was conducted to identify variables independently associated with caregiver burden. The dependent variable was the total score on the ZBI.

The hierarchical order of blocks was defined *a priori* according to the Stress Process Model (primary stressors/care demands → secondary physical strains → psychosocial vulnerability/resources) (8).

Independent variables were entered in conceptually meaningful blocks to assess incremental contributions to the explained variance (Δ*R*^2^) at each step:

- Block 1 = primary stressors: dependence, percentage of disability, years of caregiving, and weekly hours of care.
- Block 2 = secondary strains: pain intensity (VAS in head/neck, trunk, upper and lower limbs) and total physical activity (IPAQ METs).
- Block 3 = psychosocial: anxiety and depression (HADS) and the SF-36 Mental Health Subscale.
- Block 4 = QoL resources: additional SF-36 domains, including physical functioning.

Listwise deletion was applied for missing data. Model adequacy and assumptions were verified through residual diagnostics (normality, homoscedasticity, independence), Durbin–Watson statistics for autocorrelation, and collinearity diagnostics (VIF < 5, tolerance > 0.20).

##### 2.6.3.1 Mediation analysis

To determine whether caregiver mental health mediated the relationship between patient dependence and caregiver burden, a Baron and Kenny causal-step approach was applied. Four regression equations were tested sequentially to verify:

- The association of dependence on burden (path c).
- The association of dependence on mental health (path a).
- The association of mental health on burden (path b).
- The association of dependence on burden after controlling for mental health (path c′).

##### 2.6.3.2 Moderation analysis

A hierarchical regression analysis was conducted to test whether caregiver mental health moderated the association between patient dependence and caregiver burden. Both predictors were mean-centered prior to analysis. In Step 1, patient dependence and caregiver mental health were entered as main effects. In Step 2, the interaction term (Dependence × Mental Health) was introduced. A significant interaction coefficient and significant ΔR^2^ were considered evidence of moderation.

Assumptions of linearity, homoscedasticity, and normality of residuals were verified in all models.

To address potential construct overlap between caregiver burden and psychological or quality-of-life measures, multicollinearity diagnostics were carefully examined. Variance inflation factors (VIF), tolerance values, and condition indices were calculated to assess redundancy among predictors.

Given the sample size (*n* = 73), the number of predictors included in the final regression model was limited to theoretically relevant variables. The subject-to-predictor ratio (14:1) was consistent with commonly recommended guidelines for multiple regression analyses. To reduce risk of overfitting, predictors were entered hierarchically based on theoretical relevance, and multicollinearity diagnostics (VIF, tolerance, condition index) were examined.

## 3 Results

### 3.1 Sociodemographic and clinical characteristics

A total of 73 informal caregivers participated in the study. The mean age of caregivers was 61.1 ± 13.6 years, and the average weekly caregiving time was 82.2 ± 52.7 h. The mean functional dependence of the care recipient was 2.75 ± 0.7, indicating a moderate level of dependence. The mean ZBI total score was 38.6 ± 13.6, suggesting a moderate-to-high caregiver burden. The average mental health component of the SF-36 was 60.7 ± 19.9, reflecting an overall satisfactory mental well-being. Table 1 details the sociodemographic characteristics of the sample.

**Table 1:** Sociodemographic characteristics.

Variable	Mean (SD)	Count (%)
Caregiver age (years)	61.10 (13.56)	
Time spent caring for the patient (years)	16.11 (14.86)	
Time spent caring for the patient (hours per week)	82.17 (52.65)	
Patient age (years)	52.27 (29.23)	
Caregiver sex (female)		51 (69.9%)
Caregiver’s marital status		
Single		9 (12.3%)
Married		57 (78.1%)
Divorced		5 (6.8%)
Widowed		2 (2.7%)
Physical treatment last 3 months (yes)		25 (34.3%)
% of disability*		
<33%		13 (17.8%)
33–65%		7 (9.5%)
>65%		53 (72.6%)
Dependency grade*		
None		2 (2.7%)
Moderate		3 (4.1%)
Severe		6 (8.2%)
Deep		60 (82.2%)

SD, statistical deviation. *Disability percentage and dependency grade were caregiver-reported based on the most recent official social services assessment/resolution under the Spanish dependency framework.

### 3.2 Relationships between variables

Spearman correlation analyses revealed significant associations between caregiver burden and several independent variables. Higher burden scores were correlated with poorer mental health (*r* = −0.55, *p* < 0.001), and higher anxiety (*r* = 0.48, *p* < 0.001) and depression (*r* = 0.34, *p* = 0.003) scores. In addition, greater musculoskeletal pain intensity was associated with higher levels of perceived burden with a statistical significance of *p* < 0.05 in all regions evaluated. These findings indicate that both physical and psychological dimensions are relevant contributors to caregiver stress. However, no statistically significant correlation was observed between patient dependence and caregiver burden.

### 3.3 Hierarchical regression analysis

After conducting descriptive analyses, hierarchical multiple regression models were performed with the ZBI score as the dependent variable.

#### 3.3.1 Block 1—patient-related factors

Dependence, disability, years of caregiving, and weekly caregiving hours were entered first. The model yielded *R* = 0.443, *R*^2^ = 0.196, and adjusted *R*^2^ = 0.067 (*p* = 0.225). None of the individual variables showed statistical significance at this stage, although patient dependence showed a trend toward relevance in subsequent steps.

#### 3.3.2 Block 2—pain and physical activity

The addition of caregiver pain (VAS by region) and total physical activity (METs) substantially improved model fit (*R* = 0.751, *R*^2^ = 0.565, Δ*R*^2^ = 0.369, Δ*F* = 3.387, *p* = 0.022). Within this block, trunk/back pain (*β* ≈ −0.49, *p* = 0.031) and lower-limb pain (*β* ≈ 0.40, *p* = 0.043) showed statistically significant associations with caregiver burden, whereas METs did not contribute significantly.

#### 3.3.3 Block 3—psychological factors

When adding anxiety (HADS-A), depression (HADS-D), and mental health (SF-36), the model showed *R* = 0.795, *R*^2^ = 0.632, and Δ*R*^2^ = 0.068 (Δ*F* = 1.043, *p* = 0.399). Although the global increase in explained variance was not significant, SF-36 Mental Health and HADS-Anxiety emerged as relevant independent associations in the multivariable context.

#### 3.3.4 Block 4—quality of life extension

Incorporating the remaining SF-36 domains yielded the final model (*R* = 0.851, *R*^2^ = 0.725, global *p* < 0.001). The incremental change was not statistically significant (Δ*F* ≈ 0.828, *p* > 0.05). In the final model, poorer mental health (SF-36 Mental Health; *β* = −0.396, *p* = 0.002) and higher anxiety (HADS-A; *β* = 0.243, *p* = 0.049) remained statistically significantly associated with caregiver burden, while patient dependence approached significance (*β* = 0.185, *p* = 0.064).

No issues of multicollinearity were detected (VIF = 1.0–3.4). Examination of residuals confirmed the assumptions of normality and independence (histogram and P–P plots acceptable; Durbin–Watson = 2.05–2.27) (see Table 2).

**Table 2:** Hierarchical multiple regression analysis predicting caregiver burden (ZBI score).

Block	*R*	*R* ^2^	Adjusted *R*^2^	Δ*R*^2^	Δ*F* (*p*)
Patient-related factors	0.443	0.196	0.067	—	— (*p* = 0.225)
Pain and physical activity	0.751	0.565	0.489	0.369	3.387 (0.022)
Psychological factors	0.795	0.632	0.553	0.068	1.043 (0.399)
Quality-of-life extension (final model)	0.851	0.725	0.652	0.093	0.828 (>0.05)

Dependent variable: ZBI total score. *β* values represent standardized coefficients from the final model. Only predictors reaching statistical significance or trend-level significance (*p* < 0.10) in the final model are reported. Δ*R*^2^ and Δ*F* refer to the change from the previous block. No multicollinearity was detected (VIF range = 1.0–3.4). Residual diagnostics confirmed normality and independence of errors (Durbin–Watson = 2.05–2.27).

### 3.4 Mediation analysis

**Figure 1:**
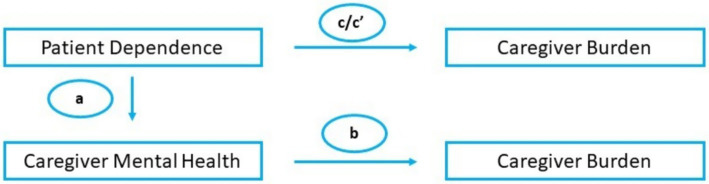
Mediation model depicting the indirect and direct effects of patient dependence on caregiver burden through caregiver mental health (paths a, b, c, and c′).

To examine whether caregiver mental health mediated the relationship between patient dependence and caregiver burden, the Baron and Kenny causal-step approach was applied (Figure 1).

Dependence was marginally associated with caregiver burden (*β* = 0.20, *p* = 0.089), indicating a weak positive relationship. However, dependence was not statistically significantly associated with caregiver mental health (*β* = −0.04, *p* = 0.713), failing to meet the prerequisite for mediation.

Mental health was strongly and inversely associated with caregiver burden (*β* = −0.54, *p* < 0.001), suggesting that poorer mental health corresponded to higher burden levels. When both variables were included in the same model, the direct effect of dependence on burden decreased slightly (*β* = 0.18, *p* = 0.075) but remained nonsignificant.

Therefore, the indirect effect through mental health was not supported by the data. The results indicate that although caregiver mental health is a strong correlate of burden, it does not act as a mediator between patient dependence and burden. Instead, mental health showed an independent association, largely explaining variance in burden irrespective of the patient’s functional status.

### 3.5 Moderation analysis

Given the absence of mediation, a moderation model was tested to explore whether caregiver mental health moderated the relationship between patient dependence and caregiver burden.

In Model 1, patient dependence and caregiver mental health were entered as main effects and explained 32.7% of the variance in caregiver burden (*R*^2^ = 0.327, *F* (2, 68) = 16.49, *p* < 0.001).

In Model 2, the interaction term (Dependence × Mental Health) was added; however, it did not significantly improve the model fit (Δ*R*^2^ = 0.024, Δ*F* (1, 67) = 2.467, *p* = 0.121). The interaction term was not statistically significant (*β* = 0.203, *p* = 0.121), indicating that caregiver mental health did not moderate the relationship between patient dependence and caregiver burden.

**Figure 2:**
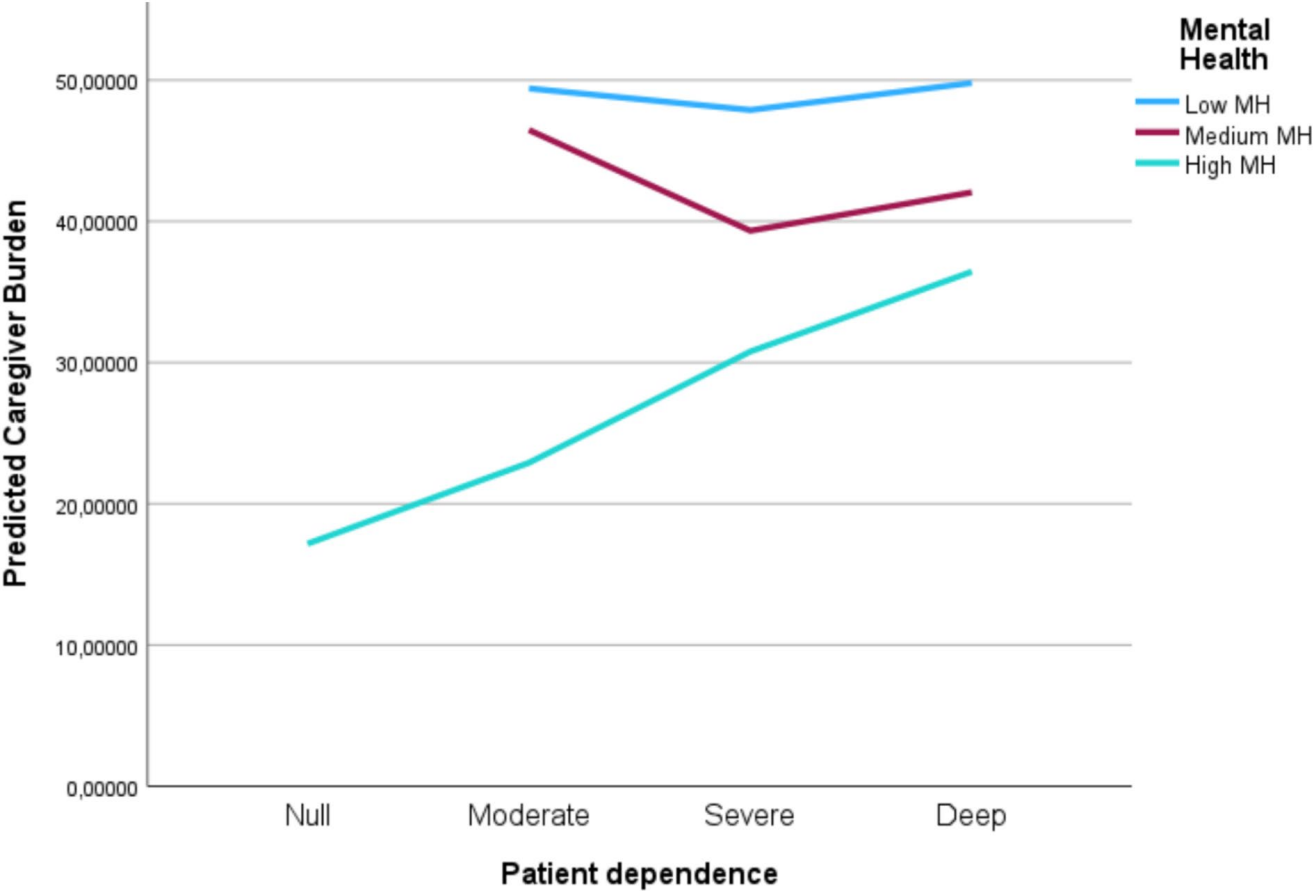
Interaction between patient dependence and caregiver mental health in predicting caregiver burden. The positive association between patient dependence and caregiver burden is stronger among caregivers with poorer mental health, and attenuated among those with higher mental health scores. MH, mental health.

Graphically (Figure 2), the slope of the dependence-burden relationship was steeper among caregivers with poorer mental health, whereas it was flatter among those with higher mental health scores.

Although the graphical pattern is suggestive of a potential buffering trend, the interaction term did not reach statistical significance.

Correlations between caregiver burden (ZBI) and psychological variables were moderate (HADS-Anxiety: *r* = 0.488; HADS-Depression: *r* = 0.342; SF-36 Mental Health: *r* = −0.553), suggesting related but non-redundant constructs. Multicollinearity diagnostics showed acceptable VIF values (range 1.026–2.407) and tolerance values above 0.40, indicating absence of problematic construct overlap. The maximum condition index was 22.17, below commonly accepted thresholds.

### 3.6 Integrated interpretation

Combining the hierarchical, mediation, and moderation analyses provides a nuanced understanding of the mechanisms underlying caregiver burden:

- Physical symptom associations: Caregiver physical pain contributes directly to burden levels, representing the objective dimension of care demands.
- Independent emotional effects: Caregiver mental health has a strong inverse association with burden, explaining substantial variance beyond physical or contextual factors.
- Moderating role of mental health: Although the interaction pattern suggested a potential buffering trend, the interaction term did not reach statistical significance.
- Explained variance and model robustness: The integrated model accounted for over 60% of the variance in caregiver burden, meeting all statistical assumptions (normality, homoscedasticity, independence of errors, and absence of multicollinearity; Durbin–Watson = 2.05).

## 4 Discussion

The findings of this study indicate that caregiver mental health and anxiety levels were the strongest independent associations of perceived burden of caring for people with dementia or cerebral palsy, followed by musculoskeletal pain, particularly in the lower back and lower limbs. In contrast, care recipient-related variables, such as the degree of dependence or disability level, were not directly associated with caregiver burden. However, this does not preclude an indirect contribution of care demands: physical strain related to daily care tasks may be more sensitively captured by caregivers musculoskeletal symptoms than by broad dependency/disability categories. These findings are consistent with recent research on caregivers of individuals with dementia and cerebral palsy, highlighting the significant influence of anxiety and depressive symptoms on the increase in caregiver burden, as well as the association of physical pain and musculoskeletal disorders on caregiver psychological well-being (28). Similarly, cross-cultural research confirms that informal caregivers experience high levels of burden, anxiety, and depression, emphasizing the central importance of mental health in caregiver well-being and resilience (29).

These results reinforce previous evidence indicating that caregiver burden is more closely linked to subjective factors, such as emotional distress or perceived self-efficacy, than to objective care-related variables (30). This suggests that the magnitude of caregiving tasks per se may be less relevant to burden than the caregiver perception and coping strategies. Caregivers with higher anxiety or depressive symptoms tend to experience caregiving as more demanding and exhausting, which may explain the strong association observed between mental health and perceived burden (31).

Although conceptual relatedness between burden and emotional distress is acknowledged, statistical diagnostics do not support construct redundancy. The moderate correlations and absence of multicollinearity indicate that caregiver burden reflects a multidimensional appraisal distinct from clinical anxiety, depressive symptomatology, or general mental health status.

Musculoskeletal pain emerged as a secondary—but significant—predictor of caregiver burden in the multivariable model, which is consistent with prior literature describing the physical consequences of prolonged caregiving, particularly when daily routines involve patient handling, assisted transfers, or mobilization (32). Importantly, because dependency and disability indicators were not directly associated with burden in our analyses, we interpret the handling/mobilization explanation as a plausible indirect pathway rather than a direct association observed in the present study. In addition, the distribution of dependency grade in our sample was highly skewed toward the highest category, which may have limited our ability to detect a direct relationship with burden. Lower back and lower-limb discomfort may reflect sustained awkward postures, lifting, and insufficient recovery, potentially exacerbating fatigue and emotional exhaustion (33).

Furthermore, the lack of a direct relationship between caregiver burden and patient-related variables, such as dependency or disability, aligns with recent research suggesting that care recipient factors primarily influence burden indirectly, through their impact on caregiver stress or perceived self-efficacy (9). These findings imply that intervention strategies should not focus solely on reducing care demands, but also on strengthening caregiver personal resources, including social support, resilience, and coping skills.

### 4.1 Clinical and scientific implications

The findings of this study highlight the pivotal role of caregiver mental health as a factor independently associated with perceived burden of caring for people with dementia or cerebral palsy, beyond the objective characteristics of caregiving, such as patient dependence or the physical demands of care. Clinically, this suggests that interventions aimed solely at improving functional outcomes in care recipients or reducing the physical load of caregiving may be insufficient to alleviate caregiver distress. Instead, a multidimensional approach that integrates psychological assessment and mental health support should be incorporated into caregiver care programs. Routine screening for anxiety and emotional well-being in informal caregivers could facilitate early identification of individuals at higher risk of burden and promote timely psychosocial or therapeutic interventions.

A multidimensional approach to caregiver support—integrating emotional, psychosocial, and practical components—has been widely emphasized in dementia care guidance and evidence syntheses (34). Therefore, we do not present this concept as novel. Instead, the contribution of the present study is to provide an integrated multivariable assessment of factors associated with caregiver burden by jointly modelling care-recipient functional severity indicators (dependency/disability) together with caregivers’ region-specific musculoskeletal pain and mental health. This framework helps identify which caregiver-related, potentially modifiable factors show the most robust associations with burden in our sample and may inform prioritization of screening and targeted support.

These implications are particularly relevant given that our caregiver profile reflects an aging caregiver population. Older caregivers may have lower physical reserve, more chronic comorbidities, and a higher risk of musculoskeletal problems, which can compound role-related strain. In practice, this supports routine screening of caregiver mental health and musculoskeletal symptoms within dementia and disability services, with clear referral pathways to psychosocial support and rehabilitation/ergonomics training (e.g., safe transfer techniques, activity pacing, and home-based exercise). From a policy perspective, older caregivers may benefit disproportionately from accessible respite services, home support, and assistive devices/home adaptations that reduce physical load. Service delivery should also consider barriers common in older adults (time constraints, mobility limitations, and potential digital exclusion), favoring flexible options (in-person/community-based programs, telephone support) and integration across health and social care.

From a scientific standpoint, the potential buffering pattern observed graphically highlights the importance of considering psychological resilience in models of caregiver strain, even though the moderation effect did not reach statistical significance. The results also support the inclusion of mental health variables as central components in exploratory models of caregiving outcomes, providing an evidence base for holistic public health policies designed to sustain informal caregiving networks and prevent caregiver burnout.

### 4.2 Limitations

This study has several limitations that should be acknowledged. First, its cross-sectional design precludes causal inferences regarding the directionality of the observed associations. Longitudinal studies are needed to determine whether poorer mental health leads to higher burden, or whether chronic burden progressively deteriorates psychological well-being. Second, the sample size was modest and limited to a specific geographical area, which may restrict the generalisability of findings to other populations or caregiving contexts. Although the sample size was modest, the number of predictors was restricted and consistent with recommended subject-to-variable ratios. Furthermore, multicollinearity diagnostics did not indicate instability of regression coefficients. Third, the absence of a control group of non-caregivers prevents comparisons that could contextualize the magnitude of caregiver-specific effects.

An additional limitation of this study is the inclusion of informal caregivers of people with dementia and of people with cerebral palsy within a single analytical framework. Although both conditions involve substantial caregiving demands, the caregiving populations may differ in terms of age, familial role, and life-course context. While our analyses focused on caregiver-related factors that cut across diagnoses, these differences may limit the generalizability of the findings to specific caregiving groups.

Unmeasured and residual confounding is also likely. Several key care-recipient indicators were caregiver-reported, including dependence grade and disability percentage (based on the most recent official social services assessment), which may introduce measurement error and attenuate associations with burden. In addition, we did not include objective clinical severity measures or standardized assessments of behavioral and cognitive symptoms (e.g., neuropsychiatric/behavioral disturbances in dementia), communication problems, or functional performance measures that are known to influence caregiver strain. Finally, other potentially relevant caregiver-level confounders were not measured (e.g., comorbidity burden, sleep quality, coping strategies, social support, and socioeconomic constraints), which limits our ability to fully adjust the multivariable models.

Finally, caregivers were recruited through two institutions using a non-probability consecutive sampling approach. Although eligibility screening and consecutive recruitment were applied within a predefined time window, caregivers connected to institutional services may differ from those not engaged with such resources, which may limit representativeness and introduces potential selection bias.

### 4.3 Future lines of research

Future research should adopt longitudinal or mixed-methods designs to explore the temporal dynamics between mental health, physical strain, and caregiver burden, thereby clarifying causal mechanisms. Interventional studies assessing the effectiveness of integrated programs that combine psychological support, stress management, and physical health promotion are warranted to confirm the clinical utility of these findings. Additionally, biomarkers of stress, sleep quality, or inflammation could also provide an objective understanding of the psychophysiological impact of caregiving. Finally, future models should examine resilience, coping strategies, and social support as potential protective factors capable of moderating the burden experienced by informal caregivers for people with dementia and cerebral palsy.

## 5 Conclusion

In this cross-sectional sample of informal caregivers of people living with dementia or cerebral palsy, caregiver mental health-related quality of life and anxiety symptoms showed the strongest independent associations with perceived burden, exceeding the associations observed for patient dependence or the physical demands of care. Poorer psychological well-being was also consistent with a stronger dependence–burden relationship. Although a buffering trend was observed graphically, moderation did not reach statistical significance.

These findings reinforce the need for multidimensional caregiver support programs that combine physical health promotion with systematic mental health assessment and accessible psychological support. Interventions addressing anxiety, stress, and emotional coping should be prioritized as core components of care strategies for caregivers of people with dementia and cerebral palsy. From a public health perspective, strengthening caregiver psychological resources may help preserve caregiver well-being and contribute to the sustainability of community-based long-term care. Given the relatively older caregiver profile in this study, implementation efforts should prioritize age-sensitive supports, including flexible service delivery and measures that reduce physical load.

## Data Availability

The raw data supporting the conclusions of this article will be made available by the authors, without undue reservation.
